# Genetic and Clinical Landscape of Chinese Frontotemporal Dementia: Dominance of *TBK1* and *OPTN* Mutations

**DOI:** 10.1002/alz70856_103146

**Published:** 2025-12-25

**Authors:** Haitian Nan

**Affiliations:** ^1^ Xuanwu Hospital, Capital Medical University, Beijing, China, Beijing, China

## Abstract

**Background:**

Our study aims to evaluate the genetic and phenotypic spectrum of Frontotemporal dementia (FTD) gene variant carriers in Chinese populations, investigae mutation frequencies, and assess the functional properties of *TBK1* and *OPTN* variants.

**Methods:**

Clinically diagnosed FTD patients underwent genetic analysis through exome sequencing, repeat‐primed polymerase chain reaction, and Sanger sequencing. *TBK1* and *OPTN* variants were biologically characterized *in vitro* using immunofluorescence, immunoprecipitation, and immunoblotting analysis. The frequencies of genes implicated in FTD in China were analyzed through a literature review and meta‐analysis.

**Results:**

Of the 410 Chinese FTD patients, 95 (23.2%) carried potential causative variants in FTD‐related genes. Among the 95 patients with gene mutations, 33 had a family history, and 62 patients are sporadic patients. The pathogenic gene results in FTD patients are as follows: 21 cases of the *MAPT* gene, 11 cases of the *GRN* gene, 10 cases of the *ANXA11* gene, 8 cases of the *TBK1* gene, 7 cases of the *SQSTM1* gene, 6 cases of the *OPTN* gene, 4 cases each of the *TARDBP*, *CHMP2B*, and *PRNP* genes, 3 cases each of the *FUS* and *NOTCH3* genes, 2 cases each of *C9orf72* repeat expansion, *CCNF*, and *VCP* genes, and 1 case each of the *HNRNPA1* gene, *HTT* repeat expansion, *AR* repeat expansion, *SPAST*, *TREM2*, *CHCHD2*, *UBQLN2*, and *TUBA4A* genes. Among them, 52 variants have not been reported in literature and can be considered novel, including the *MAPT* p.D54N, p.E342K, p.R221P, p.T263I, c.1733‐7_1733‐4del, *TBK1* p.E696G, p.I37T, p.E232Q, p.S398F, p.T78A, p.Q150P, p.W259fs, p.Thr176Arg, p.Lys197Asnfs, p.Ala492Val, *OPTN* p.R144G, p.F475V, *GRN* p.V473fs, p.C307fs, p.R101fs, c.350‐2A>C, p.Asp254Ter, p.A350V, *CHMP2B* p.K6N, p.R186Q, p.Lys130Glu, *ANXA11* p.Q155Ter, p.Arg412Trp, *CYLD* p.T157I, p.V30I, p.Val195Ala, c.1021+3A>G, *SQSTM1* p.S403A, p.V240I, p.Gly219Ser, p.Leu166Phe, p.Pro438Thr, *UBQLN2* p.P509H, *CCNF* p.S160N, p.Asp606Asn, p.Gln762Serfs, p.His735Gln, *CHCHD10* p.A8T, *SIGMAR1* p.S117L, *CHCHD2* p.P53fs, *FUS* p.S235G & p.S236G, *PRNP* c.2T>C p.Met1?, *TUBA4A* c.227‐1G>T, p.Arg339Cys, *TARDBP* p.Trp385Ser, and *TMEM106B* p.L144V variants.

**Conclusions:**

Our study demonstrates the extensive genetic and phenotypic heterogeneity of Chinese FTD patients. *MAPT, GRN*, and *TBK1* mutation carriers are the top three in Chinese FTD patients.

